# Favorable clinical outcome with intravitreal aflibercept treatment in a case with bilateral choroidal neovascular membrane and quiescent Vogt-Koyanagi-Harada syndrome

**DOI:** 10.3205/oc000150

**Published:** 2020-04-17

**Authors:** Sefik Can Ipek, Ziya Ayhan, Sinan Emre, Ali Osman Saatci

**Affiliations:** 1 Department of Ophthalmology, Dokuz Eylul University, Izmir, Turkey; 2 Department of Ophthalmology, Baskent University Zubeyde Hanim Hospital, Izmir, Turkey

**Keywords:** aflibercept (862111-32-8), choroidal neovascularization, posterior uveitis, Vogt-Koyanagi-Harada syndrome

## Abstract

**Objective:** To describe the favorable clinical outcome in a case with bilateral choroidal neovascular membrane and quiescent Vogt-Koyanagi-Harada (VKH) syndrome by administering bilateral intravitreal aflibercept injections.

**Case report:** A 30-year-old woman was diagnosed with VKH syndrome at another institution and had been in remission with oral mycophenolate mofetil for two years. However, nearly simultaneous right juxtafoveal and left subfoveal type 2 choroidal neovascular membrane was detected two years after the initial diagnosis. The right eye (OD) received three and the left eye (OS) received four aflibercept injections within a time span of eight months. Visual acuity was 20/30 in OD and 20/25 in OS at the last follow-up visit.

**Conclusion:** Although suppression of inflammation is a must in eyes with inflammatory type choroidal neovascular membranes, anti-VEGF (vascular endothelial growth factor) therapy with agents, such as aflibercept in the present case, is a key therapeutic adjunct and may possibly help improve the visual prognosis.

## Introduction

Choroidal neovascularization (CNV) may develop in eyes with various posterior uveitic entities during the course of disease. Inflammatory CNVs may develop as the result of inflammation-induced Bruch’s membrane disruption and/or the increase of several inflammatory mediators including VEGF (vascular endothelial growth factor) [1]. CNV can be a major cause of visual loss in patients with Vogt-Koyanagi-Harada (VKH) syndrome [2]. The presence of extensive retinal pigment epithelial changes was considered as a risk factor for the development of CNV [3]. With the help of fluorescein angiography (FA), Inomata et al. [4] concluded that peripapillary and macular areas were the main sites of predilection for CNV due to the concentration of inflammatory foci in those areas.

In a retrospective cohort study including 4,041 eyes of 2,307 patients with posterior uveitis and panuveitis, 81 eyes (2%) had active CNV or sequelae of past CNV at presentation. In this cohort, only one of 320 eyes with VKH syndrome had CNV (0.3%) [5]. However, in other studies CNV was noted in 8 [6] to 14.7% [7] of patients with VKH syndrome.

We hereby report a patient with VKH syndrome in remission with oral mycophenolate mofetil, who subsequently developed bilateral CNV and was treated successfully with intravitreal aflibercept injections.

## Case description

**Figure 1:**
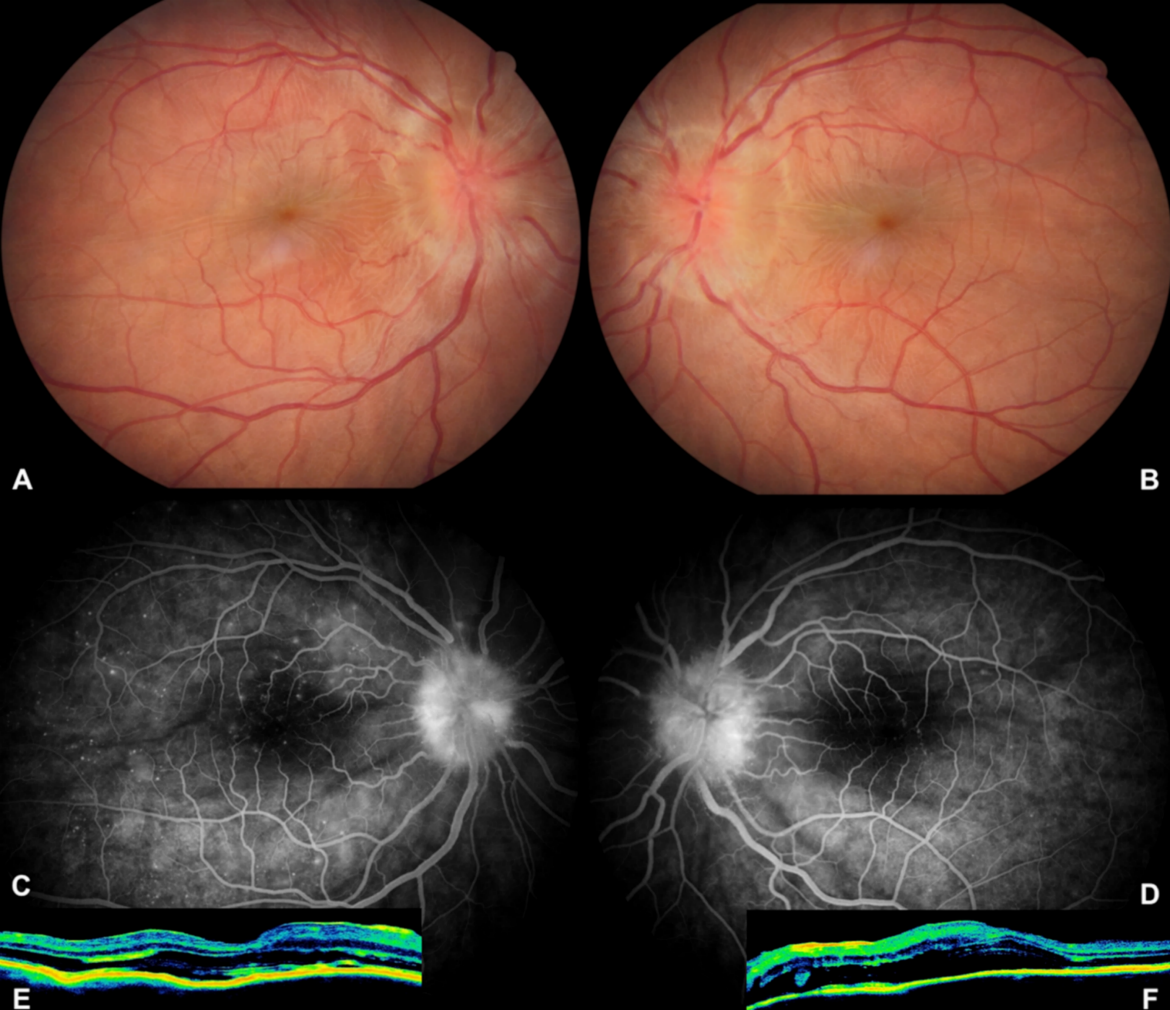
During the initial diagnosis of Vogt-Koyanagi-Harada syndrome, color fundus photographs of the right (A) and left (B) fundus show serous retinal detachment and swollen optic nerve head, venous phase of angiogram depicts fluorescein leakage from the optic disk associated with multiple pinpoint leakages at the posterior pole, (C) right and (D) left eyes; optical tomographic section delineates serous retinal detachment and corrugation of the retinal pigment epithelium/choroid layer in (E) right and (F) left eyes.

**Figure 2:**
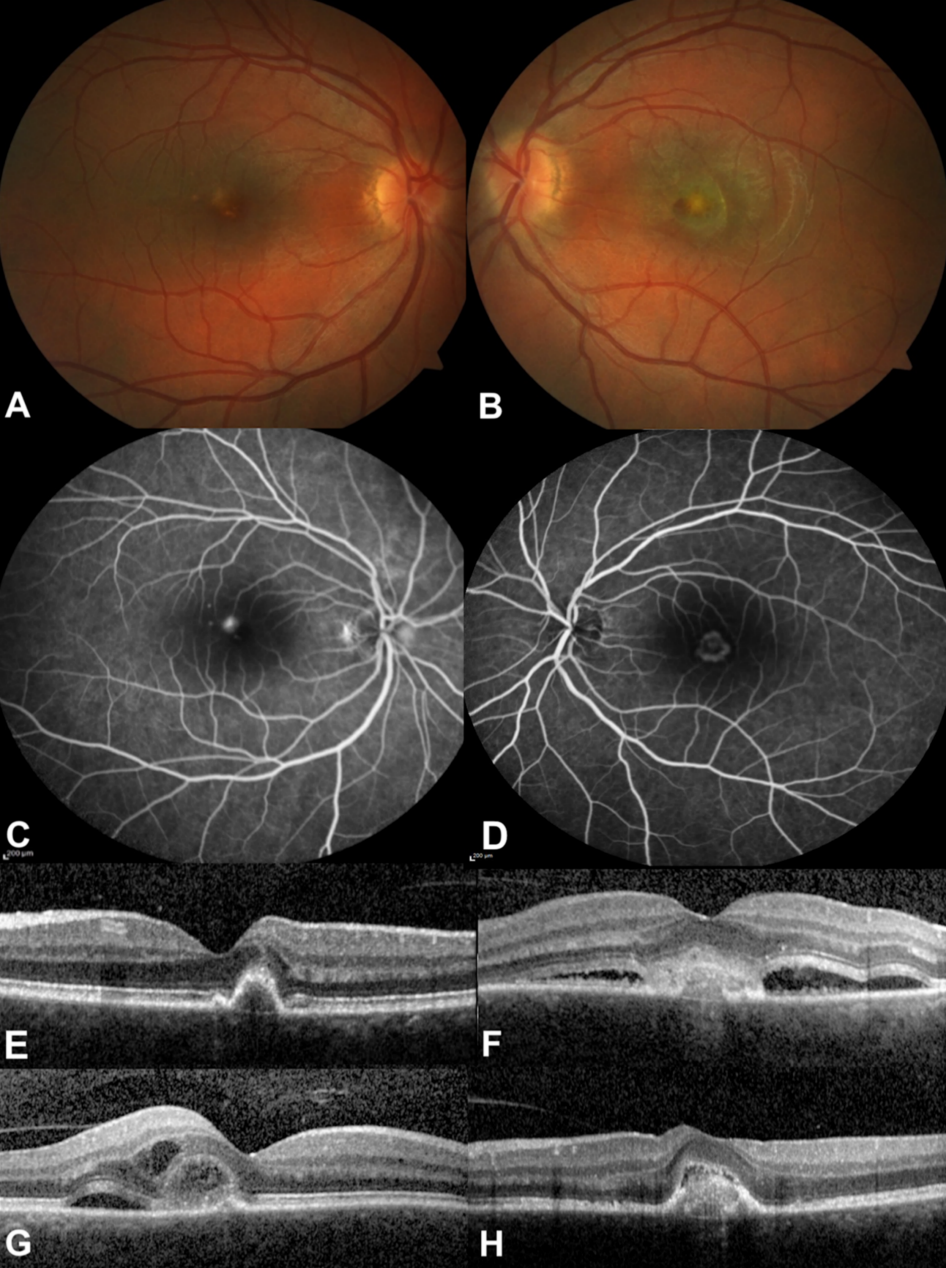
During the initial diagnosis of choroidal neovascularization (CNV), color fundus photographs of the right (A) and left (B) posterior pole show the foveal lesions, venous phase of angiogram demonstrates foveal hyperfluorescence, (C) right and (D) left eyes; optical coherence tomographic section depicts juxtafoveal (E, right eye) and subfoveal (F, left eye) CNV. A week following the left aflibercept injection, subretinal fluid greatly subsided in OS (H, left eye), but the subretinal and intraretinal fluid increased in OD just prior to aflibercept injection (G, right eye).

**Figure 3:**
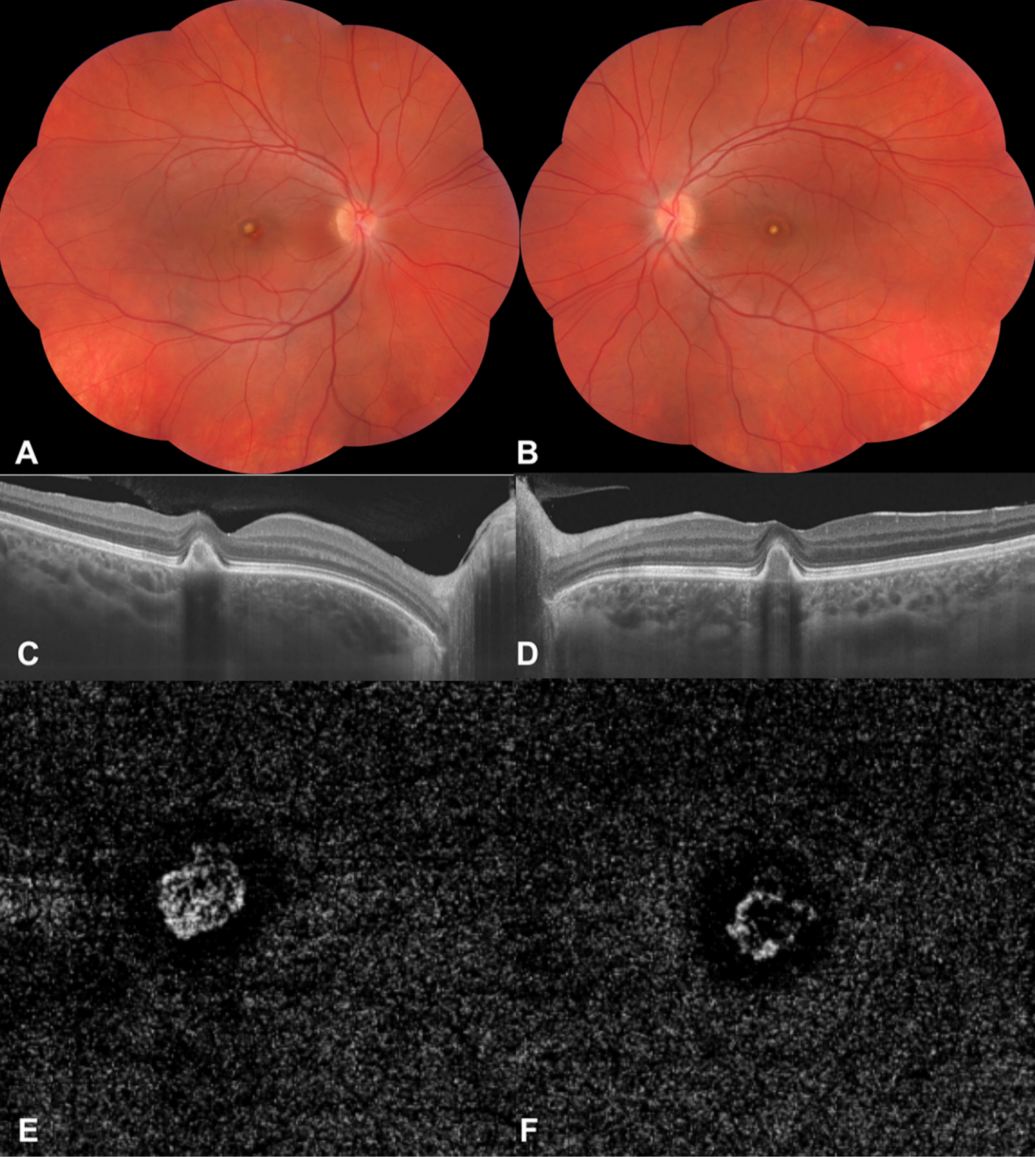
Eight months after the diagnosis of choroidal neovascularization (CNV), color fundus photographs of the right (A) and left (B) eyes show stable-looking foveas with relatively reduced CNV. Optical coherence tomographic section shows stable CNV without intraretinal and subretinal fluid, (C) right and (D) left eyes. Optical coherence tomographic angiogram (3×3, outer retinal slab) delineates the residual CNV, (E) right eye and (F) left eye.

A 30-year-old woman had been diagnosed to have VKH syndrome at another eye center two years prior and had been treated successfully with high-dose systemic steroids and then with oral mycophenolate mofetil (MMF) without any recurrence. She was referred to us due to the development of nearly simultaneous bilateral CNV while she was still on a daily maintenance dose of oral 500 mg MMF. Her color fundus photographs, fluorescein angiogram (FA), and optical coherence tomographic (OCT) examination at the initial diagnosis of VKH syndrome were obtained (Figure 1 (Fig. 1)). Upon our examination, her visual acuity was 20/25 in the right eye (OD) and 20/60 in the left eye (OS). No signs of anterior chamber inflammation were present in both eyes (OU); however, trace residual cells were present in OD. Intraocular pressure was 16 mmHg OU. Grayish looking juxtafoveal and subfoveal lesions in the OD and OS respectively were noted (Figure 2A and B (Fig. 2)). FA and OCT revealed bilateral type 2 CNV (Figure 2C, D, E, and F (Fig. 2)) without any other findings indicative of active inflammation. Initial treatment of OS was with intravitreal aflibercept. At the 1-week follow-up, visual acuity was 20/30 in OD and 20/40 in OS. Control OCT revealed that the juxtafoveal CNV was enlarged significantly with some intraretinal fluid in OD (Figure 2G (Fig. 2)) whereas the subretinal fluid in OS was mostly resolved (Figure 2H (Fig. 2)). Intravitreal aflibercept was then administered immediately to the right eye. Due to regulations for reimbursement in Turkey, all patients undergoing aflibercept or ranibizumab injections should receive three successive anti-VEGF injections four weeks apart. The patient’s OS received an additional injection eight weeks after the first three bilateral mandatory injections, due to evidence of minimal amount of intraretinal fluid, and the patient was followed up bi-monthly. Overall, OD received a total of three and OS a total of four aflibercept injections in a time frame of eight months, while the systemic treatment was continued. At the final visit, the patient’s visual acuity was 20/30 in OD and 20/25 in OS. Both macular lesions were determined stable upon clinical and OCT examinations, and no further injections were administered (Figure 3A, B, C and D (Fig. 3)). Moreover, OCT angiography pictures were obtained (Figure 3E and F (Fig. 3)).

The patient was advised to have regular bi-monthly follow-ups unless an unexpected change occurred. Overall she had a follow-up of eight months.

## Discussion

There is no clear standard of care for CNV management in inflammatory disorders. Steroids and immunosuppressives are often used in uveitis to reduce the inflammatory stimulus which seems to play a role in CNV formation, and also partly due to their antiangiogenic effect [8]. However, locally targeted therapy is often administered to treat the CNV in order to achieve an anatomically and visually superior outcome [1], [9], [10].

Currently, anti-VEGF injections have replaced photodynamic therapy (PDT) as the main treatment modality for CNVs [11]. There are a few reports on intravitreal bevacizumab and ranibizumab treatment in eyes with CNV related to VKH syndrome [12], [13], [14], [15], [16], [17]. Wu et al. [12] reported on two patients with VKH syndrome and CNV who were treated with intravitreal bevacizumab injections. One of the patients had an extrafoveal CNV, and therefore received four bevacizumab injections over 11 months in combination with laser photocoagulation. The second patient had a subfoveal CNV that responded well to a single bevacizumab injection with a follow-up of seven months.

Mansour et al. [13] evaluated eight patients with inflammatory CNVs refractory to standard therapy. They were treated with intravitreal bevacizumab injections and were followed up for five years after the first injection. Two of the patients had VKH syndrome, and combined juxtapapillary and peripapillary CNV. One patient received a single bevacizumab injection and the other received 15 bevacizumab injections, both with a relatively good visual outcome. D’Souza et al. [14] reported on a series of 15 consecutive patients with inflammatory CNVs treated with intravitreal bevacizumab injections. Two of those patients had VKH syndrome, and both patients had peripapillary CNV and received only a single injection of bevacizumab. One patient had a follow-up of six months and the other of 12 months. Pai et al. [15] administered a combination of triamcinolone and bevacizumab twice in an eye with active panuveitis and peripapillary CNV in a patient with VKH syndrome six months apart with a favorable visual outcome. Kolomeyer et al. [16] reported on a VKH patient who had undergone methotrexate treatment and developed unilateral parafoveal CNV. The eye was then treated with four ranibizumab injections during a time frame of 12 months. The visual outcome was unfortunately poor. In a very recent study, Sakata et al. [18] evaluated the outcome of intravitreal bevacizumab administration in seven eyes of six patients with CNV and VKH syndrome. The CNV was juxtapapillary in two eyes, juxtafoveal in three eyes and subfoveal in two eyes. Two eyes had active inflammation. Five patients (five eyes) completed a 12-month follow-up and received 12 bevacizumab injections. Visual acuity was improved in four out of five eyes. However, all cases required either introduction or increment of immunosuppressive therapy.

Anti-VEGF treatment protocol is still somewhat uncertain in inflammatory CNVs. A randomized study investigating the efficacy and safety of ranibizumab for the treatment of CNV due to uncommon causes of CNV (i.e. other than age-related macular degeneration and degenerative myopia) revealed that in a group of 178 patients [19], 18 (15.1%) had post-inflammatory type CNV. A single loading dose followed by pro re nata regimen seemed to succeed statistically both in anatomic and visual outcomes. There was a treatment effect of 6.5 letters at two months after the ranibizumab injection.

In the present case, a patient with quiescent VKH syndrome without any sign of active inflammation presented with bilateral type 2 CNV (one juxtafoveal and the other subfoveal) despite being in a state of remission. We administered three aflibercept injections in OD and four aflibercept injections in OS during an eight-month period as mentioned in the case description above. Concurrently, the patient maintained the ongoing therapy of oral mycophenolate mofetil. Due to a lack of comparative studies, advantage of administering aflibercept over bevacizumab or ranibizumab on inflammatory CNVs is not clear. To our present knowledge, aflibercept administration has not been reported before in CNV related to VKH syndrome. Clinicians should be cautious in differentiating the signs of CNV from the signs of pre-existing uveal inflammation in eyes with various uveitic entities, as the original disease can be active or in remission.

## Conclusion

Anti-VEGF agents (aflibercept administrations in the present case) appear to be a very valuable adjunct to obtain favorable anatomic and visual outcomes in most of the eyes of patients with VKH syndrome and coexistent CNV.

## Notes

### Competing interests

The authors declare that they have no competing interests.

### Ethical statement

Written informed consent was obtained from the patient for the publication of this case report.

## References

[1] Battaglia Parodi M, Iacono P, Verbraak FD, Bandello F (2010). Antivascular endothelial growth factors for inflammatory chorioretinal disorders. Dev Ophthalmol.

[2] Moorthy RS, Inomata H, Rao NA (1995). Vogt-Koyanagi-Harada syndrome. Surv Ophthalmol.

[3] Moorthy RS, Chong LP, Smith RE, Rao NA (1993). Subretinal neovascular membranes in Vogt-Koyanagi-Harada syndrome. Am J Ophthalmol.

[4] Inomata H, Minei M, Taniguchi Y, Nishimura F (1983). Choroidal neovascularization in long-standing case of Vogt-Koyanagi-Harada disease. Jpn J Ophthalmol.

[5] Baxter SL, Pistilli M, Pujari SS, Liesegang TL, Suhler EB, Thorne JE, Foster CS, Jabs DA, Levy-Clarke GA, Nussenblatt RB, Rosenbaum JT, Kempen JH (2013). Risk of choroidal neovascularization among the uveitides. Am J Ophthalmol.

[6] Wu K, Zhang X, Su Y, Ji Y, Zuo C, Li M, Wen F (2016). Clinical Characteristics of Inflammatory Choroidal Neovascularization in a Chinese Population. Ocul Immunol Inflamm.

[7] Lertsumitkul S, Whitcup SM, Nussenblatt RB, Chan CC (1999). Subretinal fibrosis and choroidal neovascularization in Vogt-Koyanagi-Harada syndrome. Graefes Arch Clin Exp Ophthalmol.

[8] Bhende M, Ahmed AS (New). Management of Inflammatory CNV. In: Uveitis: An Update.

[9] Saatçi AO, Ayhan Z, İpek ŞC, Söylev Bajin M (2018). Intravitreal Aflibercept as an Adjunct to Systemic Therapy in a Case of Choroidal Neovascular Membrane Associated with Sympathetic Ophthalmia. Turk J Ophthalmol.

[10] Saatci AO, Ayhan Z, Engin Durmaz C, Takes O (2015). Simultaneous Single Dexamethasone Implant and Ranibizumab Injection in a Case with Active Serpiginous Choroiditis and Choroidal Neovascular Membrane. Case Rep Ophthalmol.

[11] O’Keefe GA, Rao NA (2017). Vogt-Koyanagi-Harada disease. Surv Ophthalmol.

[12] Wu L, Evans T, Saravia M, Schlaen A, Couto C (2009). Intravitreal bevacizumab for choroidal neovascularization secondary to Vogt-Koyanagi-Harada syndrome. Jpn J Ophthalmol.

[13] Mansour AM, Mackensen F, Mahendradas P, Khairallah M, Lai TY, Bashshur Z (2012). Five-year visual results of intravitreal bevacizumab in refractory inflammatory ocular neovascularization. Clin Ophthalmol.

[14] D’Souza P, Ranjan R, Babu U, Kanakath AV, Saravanan VR (2018). INFLAMMATORY CHOROIDAL NEOVASCULAR MEMBRANE: Long-Term Visual and Anatomical Outcomes After Intravitreal Anti-vascular Endothelial Growth Factor Therapy. Retina.

[15] Pai SA, Hebri SP, Lootah AM (2012). Management of recurrent inflammatory choroidal neovascular membrane secondary to Vogt-Koyanagi-Harada syndrome, using combined intravitreal injection of bevacizumab and triamcinolone acetate. Indian J Ophthalmol.

[16] Kolomeyer AM, Roy MS, Chu DS (2011). The use of intravitreal ranibizumab for choroidal neovascularization associated with Vogt-Koyanagi-Harada syndrome. Case Rep Med.

[17] Arevalo JF, Adan A, Berrocal MH, Espinoza JV, Maia M, Wu L, Roca JA, Quiroz-Mercado H, Ruiz-Moreno JM, Serrano MA, Pan-American Collaborative Retina Study Group (2011). Intravitreal bevacizumab for inflammatory choroidal neovascularization: results from the Pan-American Collaborative Retina Study Group at 24 months. Retina.

[18] Sakata VM, Morita C, Lavezzo MM, Rodriguez EEC, Abdallah SF, Pimentel SLG, Hirata CE, Yamamoto JH (2019). Outcomes of Intravitreal Bevacizumab in Choroidal Neovascularization in Vogt-Koyanagi-Harada Disease – A Prospective Study. Ocul Immunol Inflamm.

[19] Lai TYY, Staurenghi G, Lanzetta P, Holz FG, Melissa Liew SH, Desset-Brethes S, Staines H, Hykin PG, MINERVA study group (2018). Efficacy and safety of ranibizumab for the treatment of choroidal neovascularization due to uncommon cause: Twelve-Month Results of the MINERVA Study. Retina.

